# Gasless transvaginal natural orifice transluminal endoscopic surgery for hysterectomy and salpingectomy on a robot platform with flexible devices in a porcine model

**DOI:** 10.1038/s41598-024-55576-7

**Published:** 2024-03-04

**Authors:** Youwen Mei, Yanjun Wang, Qiang Zhang, Liling Xiong, Li Xu, Qiannan Hou, Jiaojiao Chen, Li He, Yonghong Lin

**Affiliations:** Department of Gynaecology and Obstetrics, Chengdu Women’s and Children’s Central Hospital, School of Medicine, University of Electronic Science and Technology of China, Chengdu, 611731 China

**Keywords:** V-NOTES, Robotic surgical procedures, Gasless technique, Porcine, Animal model, Urogenital reproductive disorders, Reproductive disorders

## Abstract

In this report, we described a new technique of gasless V-NOTES for hysterectomy and salpingectomy on a robotic platform with flexible devices in a porcine model. As a result, the gynecological procedures were successfully completed. The total operative time was 110 min, while the docking time was 10 min. The estimated blood loss was estimated to be 10 mL with no intraoperative complications. It revealed that gasless V-NOTES for hysterectomy and salpingectomy on a robotic platform with flexible devices appeared to be feasible and safe in the porcine model and has the potential for clinical use in human beings.

## Introduction

Laparoscopy initiated the era of minimally invasive surgery, while natural orifice transluminal endoscopic surgery (NOTES) was a further step to the field. The vagina is the most widely used natural channel^1^. Compared with conventional laparoscopy, transvaginal natural orifice transluminal endoscopic surgery (v-NOTES) was preferred by surgeons as it provides better access to pelvic organs, avoids the abdominal scar, even decreases the operation time and estimated blood loss^2^. However, many barriers still stand on its way because of limited manipulators freedom and narrow operating space. In this case, robotic laparoscopy could play to its strength as it provides accurate and fine surgical procedures^3^. Although traditional robotic V-NOTES was proven to be non-inferiority compared to traditional laparoscopy^4,5^, challenge still existed as there was significant arm collisions due to space confinement and rigid-link instrumental arms^6^. For this reason, robotic platform with flexible devices was developed^7^. Currently, there were few reports published about the application of V-NOTES for hysterectomy in the robotic platform with flexible devices. Therefore, we tried to evaluate its feasibility in the present study. As a porcine was more representative to evaluate on the surgical field and assess the workspace availability in human beings, we performed this operation in a porcine model. Furthermore, we combined gasless technique in this operation to alleviate the interference of smoke generated in the surgery. We believe that the surgical experience was valuable for its application in human beings.

## Methods

**Figure 1:**
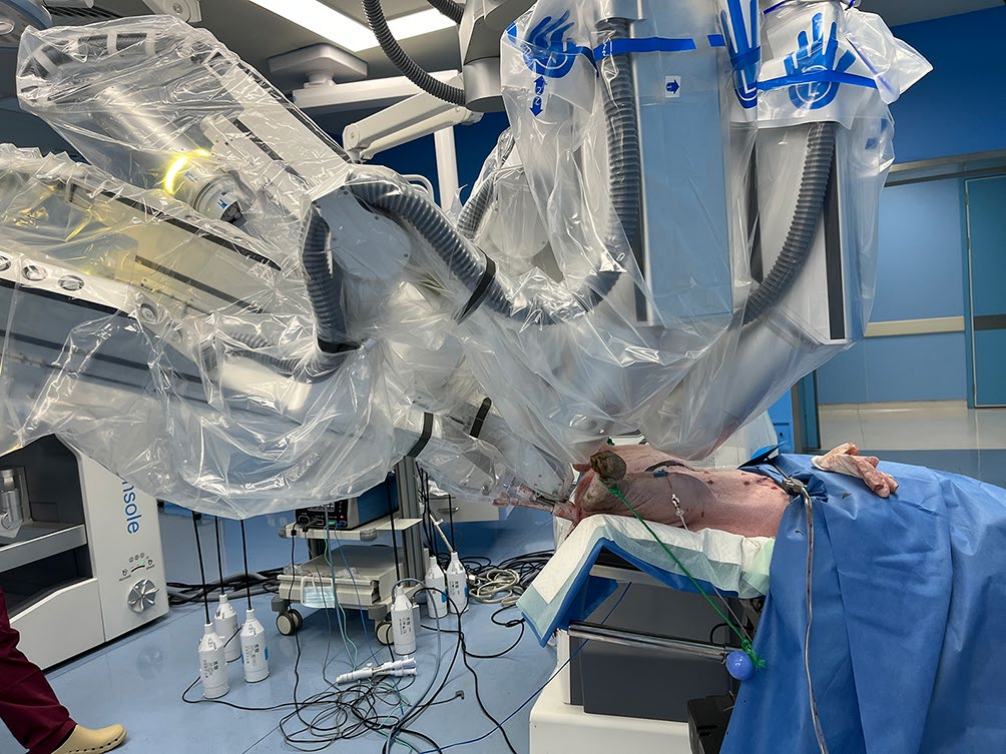
Porcine setup with the inserted cannula.

One female adult pig weighed 60 kg was used in this study. It was initially administered by 1 mg Shutai per kg for anesthesia induction and 1.5–2.5% isoflurane for maintenance. The pig was placed in supine position with low head high hip (Fig. 1).

**Figure 2:**
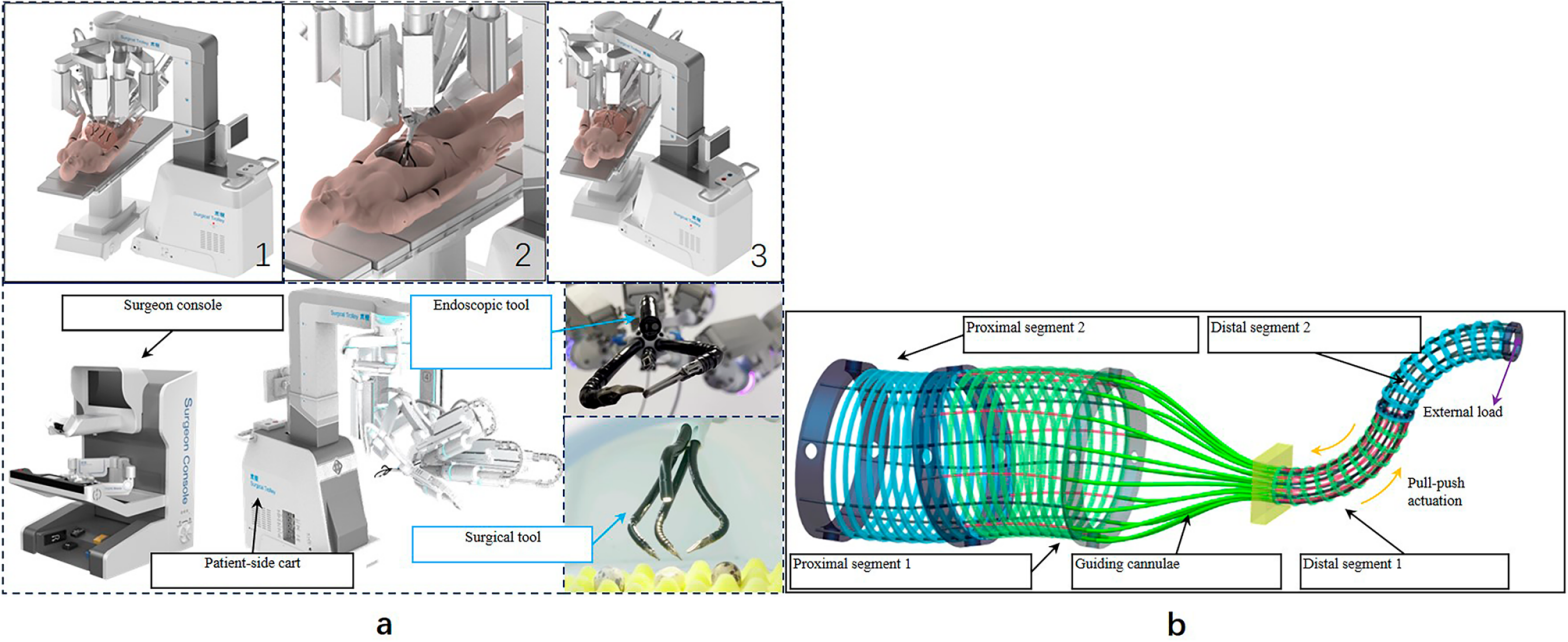
(**a**) An overview of the SHURUI endoscopic surgical robotic system. The patient-side cart can be arranged to deploy the surgical/endoscopic tools in the (1) multi-port, (2) hybrid-port, and (3) single-port configurations. (**b**) Dual continuum mechanism.

The robotic platform used in this manuscript is SHURUI system. The surgical manipulators of the SHURUI endoscopic surgical robotic system can configured for multi-port, single-port, and hybrid-port procedures (Fig. 2a). Both the surgical robotic tool and endoscopic tool in the SHURUI endoscopic surgical system are composed of so-called dual continuum mechanism. Unlike the conventional instruments that are composed of joints and rigid links, the continuum mechanism used in the SHURUI endoscopic surgical robotic system achieves its motion via continuous deformation of the elastic structure (Fig. 2b).

The surgical technique is as follows.

**Figure 3:**
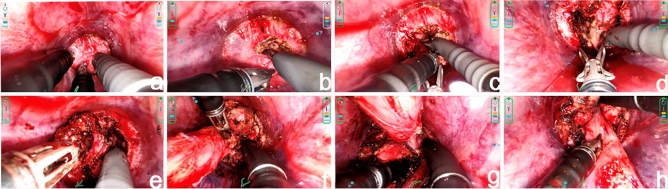
(**a**) Exposure of the cervix. (**b**) Incision of vaginal mucosa circularly. (**c**) Dissection of vaginal mucosa anteriorly and (**d**) posteriorly. (**e**) Ligation of bilateral uterine arteries. (**f**) Dissection of para-uterine tissues. (**g**) Incision of utero-rectal peritoneal reflection (**h**) and utero-vesical peritoneal reflection.

**Figure 4:**
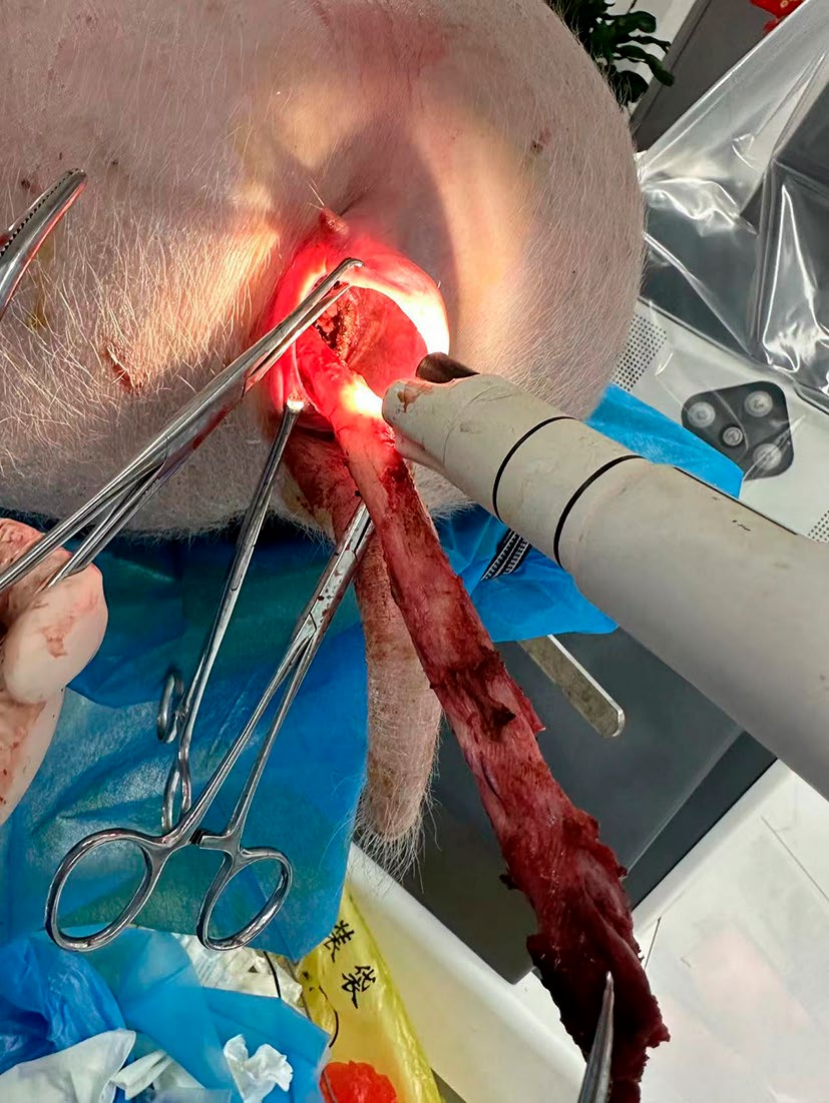
The uterus was pulled out from the vagina.

1. The vagina and cervix were exposed with a self-made cylinder (plastic cylindrical, hollow device, 5 cm in length) after disinfection. The robotic platform with flexible devices (SHURUI, China) was established (Fig. 3a).
2. The cervix was clamped and vaginal mucosa around the cervix was incised circularly to the cervical fascia (Fig. 3b).
3. The cervical anterior mucosa was pulled up and the vesico-cervical fascia was separated (Fig. 3c).
4. After pulling up the cervical posterior mucosa, the rectal-cervical fascia was separated subsequently (Fig. 3d).
5. The cervix was pulled up, the right and the parametrial fascia was separated to expose the left uterine artery, which was electro-coagulated and disconnected (Fig. 3e). The right uterine artery was disconnected similarly.
6. Bilateral para-uterine fascia and para-uterine blood vessels were separated and ligated (Fig. 3f).
7. The cervix was pulled downwards, and the utero-rectal peritoneal reflection was carefully exposed and incised (Fig. 3g). After the cervix was pulled upwards, the utero-vesical peritoneal reflection was also exposed and incised (Fig. 3h).
8. The uterus was pulled out (Fig. 4) and removed subsequently.

All surgical procedures in the porcine model were performed by the primary surgeon via a remote console (Sup Fig. 1). During the surgery, the smoke and blood were sucked away by the assistant to ensure a clear operative field. Video 1 and Video 2 shows the surgical procedure of separation of paracervical tissues and uterine bladder space. This study was reviewed and approved by [Ethics Committee of Chengdu Women and Children’s Central Hospital], with the approval number: [202378], and was carried out in accordance with the Guidelines for Research with Experimental Animals of the University of Electronic Science and Technology of China. This study was performed in compliance with the ARRIVE guidelines.

## Results

Hysterectomy was successfully completed in the porcine model. The docking time of the robotic system was 10 min. The time required hysterectomy was approximately 110 min. The blood loss for the procedure was estimated to be 20 mL, and the porcine survived the surgery without any intraoperative complications.

## Discussion

To the best of our knowledge, the study may be the first to describe the technique of gasless V-NOTES for hysterectomy and salpingectomy in the porcine model on a robot platform with flexible devices. In 2013 and 2017, Bazzi and Katagiri described a case of V-NOTES for partial nephrectomy and liver resection in a porcine model respectively^8,9^. However, it is not necessary to do the intricate dissection of para-cervical and para-uterine tissues in both cases. In 2021, Chan reported two cases of hysterectomy in the porcine model on a robot platform^6^. In this article, the surgery was performed by transrectal route, which was different from the transvaginal route in the present study. As hysterectomy was performed trans-vaginally in human beings, the surgical experience in the present study may be more applicable for human beings. Moreover, as the vaginal channel was narrower and more curved in the porcine model than human beings, it may be easier to complete surgical procedure in human beings compared with that of the porcine model.

**Figure 5:**
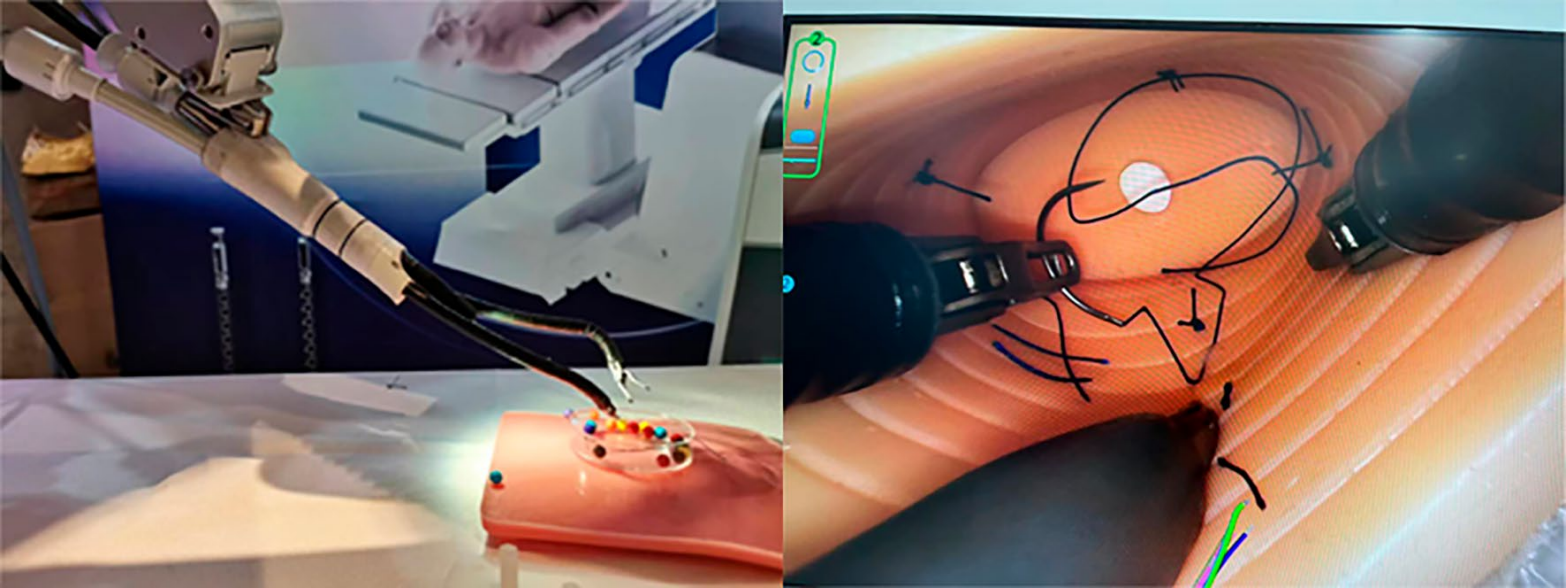
laparoscopic surgical skills training.

According to our experience, the following aspects are critical to the success of this operation. 1) Proficient surgical skills are key to the surgery. The challenge of V-NOTES is the limited maneuverability. Therefore, the surgeons should have repeated training of the surgical technique, such as gripping, suture and knotting in a confined space (Fig. 5). 2) A clear surgical vision was an important guarantee. As we know, smoke generated during the surgery led to unclear surgical vision, especially when it was performed in an enclosed space. Therefore, we adopted the gasless technique which has been proven effective in our hospital^5^. According to our experience and previous studies, gasless technique not only decreased the complications associated with CO2 pneumoperitoneum, but also reduced the operation difficulty and cost^10,11^**.** There may be concerns about the limited exposure of surgical space in gasless surgery. However, this may be resolved with our invented suspension needle by hooking up the mesocolic band or peritoneum^12^. 3) The self-made device which was placed in the vagina was helpful to open the vaginal wall. We also made a similar device compatible with the vagina of human beings which has been proven effective in our clinical practice (Sup. Fig. 2).

## Conclusion

In conclusion, gasless V-NOTES for hysterectomy and salpingectomy on the robotic platform with flexible devices in the porcine model was feasible and safe. And we believe it could be successfully applied in human beings in the near future. Of course, there are anatomic differences between porcine and human beings, further studies about its application in human beings are required.

### Supplementary Information

Supplementary Video 1.

Supplementary Video 2.

Supplementary Figure 1.

Supplementary Figure 2.

## Data Availability

The datasets used and/or analyzed during the current study available from the corresponding author on reasonable request.
