# Supplementary figures and images for: Gasless transvaginal natural orifice transluminal endoscopic surgery for hysterectomy and salpingectomy on a robot platform with flexible devices in a porcine model
Source: Sci Rep. 2024 Mar 4;14:5366. doi: 10.1038/s41598-024-55576-7 (PMC10912522; doi:10.1038/s41598-024-55576-7)

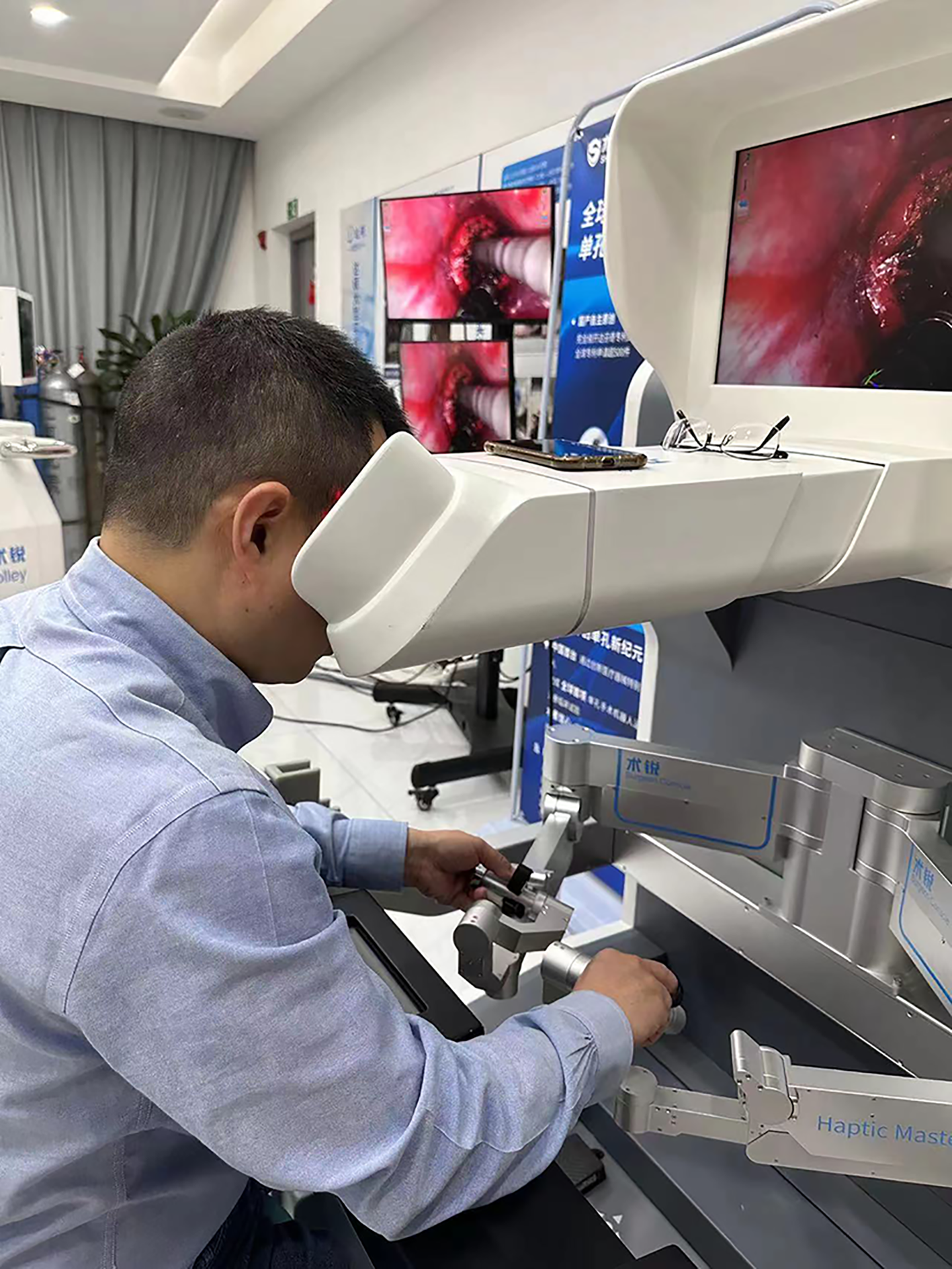

Supplement: Supplementary file 3 — Supplementary Figure 1. [file 41598_2024_55576_MOESM3_ESM.tif]

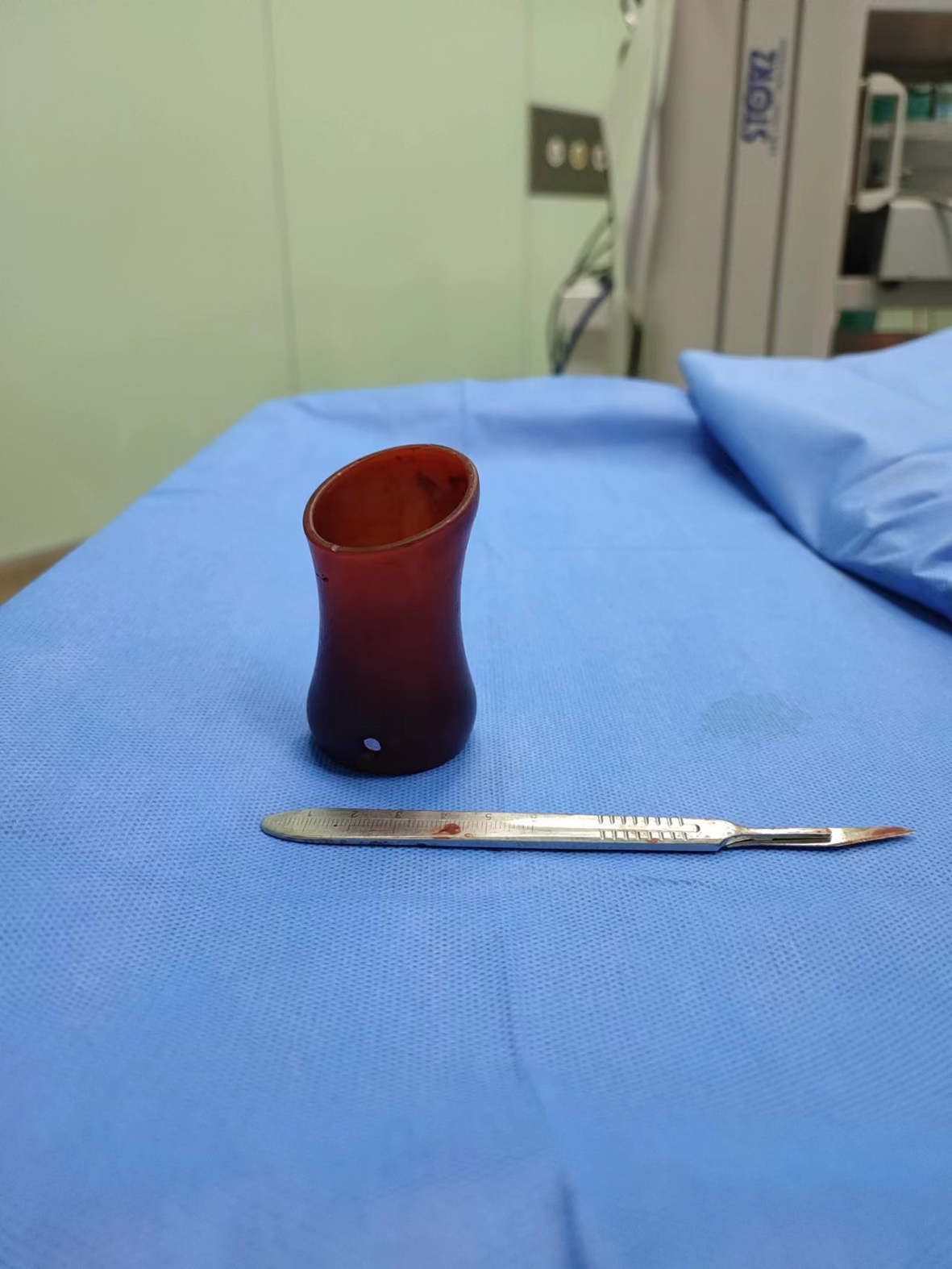

Supplement: Supplementary file 4 — Supplementary Figure 2. [file 41598_2024_55576_MOESM4_ESM.tif]
